# High-dose glucocorticoid before hip and knee arthroplasty: To use or not to use—that’s the question

**DOI:** 10.1080/17453674.2018.1475177

**Published:** 2018-05-21

**Authors:** Henrik Kehlet, Viktoria Lindberg-Larsen

**Affiliations:** 1 Section of Surgical Pathophysiology, Rigshospitalet, Copenhagen University;; 2 The Lundbeck Foundation Centre for Fast-track Hip and Knee replacement, Copenhagen, Denmark

During the last decade, “fast-track” or “enhanced recovery” programs have been introduced in hip and knee arthroplasty (THA/TKA) with a continuing improvement in recovery and consequently reduction in length of stay (LOS) (Zhu et al. 2017). Importantly, LOS may depend on early organ dysfunction, appearance of complications, or organizational factors. Early postoperative organ dysfunction including pain, orthostatic intolerance, cardiovascular and thromboembolic morbidity, cognitive disturbances, nausea and vomiting, sleep disturbances, fatigue, and loss of muscle function may all depend on factors involved in the global surgical stress response. Two main mechanisms include the neuro-endocrine responses and the inflammatory-immunological responses, where the latter may be most important to determine post THA/TKA recovery (Gaudilliere et al. 2014). Of the various pharmacological interventions to reduce the inflammatory responses, glucocorticoids are the most powerful (de la Motte et al. 2014, Steinthorsdottir et al. 2017, Toner et al. 2017). Notably, systemic perioperative glucocorticoid administration has been the subject of 7 systematic reviews and/or meta-analyses in THA/TKA since December 2016, summarizing that glucocorticoids may reduce nausea and vomiting as well as acute early postoperative pain. Interestingly, all of the 7 reviews (Hartman et al. 2017, Li et al. 2017, Liu et al. 2017, Meng and Li 2017, Yue et al. 2017, Li et al. 2018, Mohammad et al. 2018) claim to be the first and even more interestingly they include between 4 and 14 studies, thereby questioning the search methodology. Although all reviews agree that additional glucocorticoid may provide more analgesia, a critical assessment of efficacy was not done in relation to basic anesthetic/analgesic principles with different regimes in the different RCTs and a dose-finding analysis was only discussed in one review, but was not possible (Yue et al. 2017). Overall, most pain studies have used a limited dose of glucocorticoid (about 6–12 mg dexamethasone), but the most detailed studies discussed below have used a higher dose (125 mg methylprednisolone/∼ 25 mg dexamethasone). Nevertheless, a more critical update on the use of perioperative glucocorticoid in THA and TKA may be appropriate in the light of more recent findings of specific pathophysiological responses to surgery and safety aspects.

## Surgical pathophysiology

The anti-inflammatory effects of high-dose systemic glucocorticoid are well documented by reduced responses in IL-6 and CRP (Yue et al. 2017). Consequently, there seems to be less postoperative fatigue (Lunn et al. 2011, 2013), which may provide an excellent start to postoperative recovery combined with the documented analgesic and anti-nausea and vomiting effects. Recently, other interesting effects on postoperative THA/TKA responses have shown that the usual degradation of endothelial barrier function after TKA was attenuated by preoperative high-dose (125 mg) methylprednisolone (∼ 25 mg dexamethasone) (Lindberg-Larsen et al. 2017b) while there was no effect on the usual early (48 h) pronounced loss of quadriceps function (Lindberg-Larsen et al. 2017a), despite an inhibition of the inflammatory response. In addition, the endogenous anti-inflammatory protein pentraxin-3 (PTX-3) was significantly increased by preoperative high-dose glucocorticoids (Lindberg-Larsen et al. 2018b), although the clinical consequences regarding a potential reduction of infectious complications remain to be studied. The conventional effect of glucocorticoid on glucose homeostasis has also been assessed in a recent very detailed study on the different components of glucose homeostasis (hyperglycemia, insulin resistance, C-peptide response) and showed a transient impairment in glucose homeostasis lasting for only about 24–48 hours after 125 mg preoperative methylprednisolone (Lindberg-Larsen et al. 2018a). The surgery-induced increased thrombogenic responses may also be attenuated by perioperative high-dose glucocorticoid (Yue et al. 2017), although the potential positive effects on thromboembolic complications (Li et al. 2018) need further evaluation. Other aspects of early postoperative recovery after THA and TKA such as sleep disturbances (Krenk et al. 2012), and early cognitive dysfunction (Krenk et al. 2014) have not been elucidated so far. Interestingly, in one RCT, 125 mg methylprednisolone reduced early fatigue despite subjective reports on sleep quality on the first night being reduced (Lunn et al. 2011). With regard to early mobilization, preoperative 125 mg methylprednisolone did not improve orthostatic intolerance after THA despite a reduced inflammatory response (Lindberg-Larsen et al. 2018c). Finally, the pathophysiological effects of perioperative high-dose glucocorticoid on the early recovery phase from the operating theatre to the postoperative recovery unit (PACU) and discharge to the ward may potentially shorten the PACU stay due to improved opioid-sparing analgesia and PONV reduction, but calls for future detailed PACU discharge criteria studies (Steinthorsdottir et al. 2017).

In summary, preoperative high-dose glucocorticoid may attenuate some, but not all undesirable pathophysiological responses to surgery. Consequently, detailed dose-finding studies and whether repeat dosing may further enhance recovery is required, as few and inconclusive data are available. Finally, the delicate balance between necessary and undesirable inflammatory response needs attention, since an inflammatory response is required for wound healing etc., but an exaggerated response may delay recovery (Gaudilliere et al. 2014).

## Safety aspects

Overall, there seem to be no safety issues in using a high-dose of glucocorticoid perioperatively in the surgical literature (Toner et al. 2017). However, due to the small-scale RCTs in THA/TKA (Hartman et al. 2017, Li et al. 2017, Liu et al. 2017, Meng and Li 2017, Yue et al. 2017, Li et al. 2018, Mohammad et al. 2018), there was no final conclusion with regard to safety aspects. More recently, a large prospective comparative multicenter cohort study in TKA was analyzed including about 1,500 consecutive patients after introduction of high-dose glucocorticoid vs. about 2,400 patients in the period before (Jørgensen et al. 2017). These high-volume data showed no risk signals from using a high dose (125 mg methyl­prednisolone) regarding different types of medical complications or superficial wound or deep infections. However, no conclusive data are available from insulin-dependent diabetics (Lindberg-Larsen et al. 2018a) and no safety studies are available evaluating higher doses or repeat dosing or consequences on prosthesis fixation. Based upon all 7 systematic reviews, no risk of glucocorticoid-induced psychosis has been reported.

## Future strategies and conclusions

Due to the increased interest in short-stay and outpatient THA and TKA (Vehmeijer et al. 2018), and given the above-mentioned efficacy and safety aspects, the question is whether perioperative high-dose glucocorticoid may further enhance early recovery and facilitate a safe outpatient setup. A recent study in unselected patients in the Danish government-run healthcare system (Gromov et al. 2017), and including preoperative 125 mg methylprednisolone in addition to standard multimodal analgesia, showed that for about 15% of patients undergoing THA and TKA this could be performed in an outpatient setting. However, the most interesting finding from that study was the question why the remaining 85% of patients did not recover early, again emphasizing the many early recovery problems mentioned in the introduction of our article. Thus, the major future challenge that remains to be answered is whether an individualized and potentially higher dose of glucocorticoid with more pronounced anti-inflammatory effects can be safe and further enhance early recovery and reduce organ dysfunction in these 85% of patients. Interestingly, a large safety non-RCT cohort study (Jørgensen et al. 2017) showed that the proportion of patients staying >4 days was reduced by 50% by high-dose glucocorticoid. Also, future studies should focus on specific “high-risk” pain responders like pain catastrophizers, preoperative opioid users, etc. (Gilron et al. 2018) Finally, it remains to be evaluated whether the recovery benefits are correlated with the magnitude of reduction in the inflammatory responses or whether the approach should be used only in certain “high-inflammatory” responders.

In conclusion, the previous and the more recent data suggest several major beneficial recovery effects of the anti-inflammatory effects of perioperative high-dose glucocorticoid in THA and TKA and so far without safety issues. Consequently, it may at this time be appropriate to recommend its use on a “routine” basis at least in TKA, but calling for more data from specific “risk” groups (THA, high-pain responders, diabetics, infected revisions, etc.) although the optimal dose needs to be defined. Perioperative use of high doses of glucocorticoids may therefore represent a further step to enhance recovery after “fast-track” or “enhanced recovery” THA and TKA (Kehlet 2013).

Both authors have contributed equally to the preparation of the manuscript. No conflict of interest declared.
